# Prompt Configurations for Multimodal Large Language Models in Diagnosing and Staging Osteonecrosis of the Femoral Head: Multimodel Retrospective Observational Diagnostic Study

**DOI:** 10.2196/92919

**Published:** 2026-08-10

**Authors:** Jiesheng Zhu, Xingxing Huang, Jincheng Shi, Shaoming Chen, Daosen Chen, Zhihan Gao, Yi Lu, Tao Yang, Xue Wang, Yimu Lin, Peiyu Xu, Li Li, Pei Fan

**Affiliations:** 1 Department of Orthopedics, Second Affiliated Hospital & Yuying Children's Hospital of Wenzhou Medical University, No. 109, Xueyuan West Road, Wenzhou, Zhejiang, 325000, China, 86 577 88002808, 86 577 88002823; 2 Department of Orthopedics, Yunhe People's Hospital, Lishui City, Zhejiang, China; 3 The Second School of Medicine, Wenzhou Medical University, Wenzhou, Zhejiang, China; 4 Department of Radiology, Second Affiliated Hospital & Yuying Children's Hospital of Wenzhou Medical University, Wenzhou, Zhejiang, China; 5 Key Laboratory of Pediatric Anesthesiology, Ministry of Education, Wenzhou Medical University, Wenzhou, Zhejiang, China

**Keywords:** osteonecrosis of the femoral head, artificial intelligence, multimodal large language models, radiology, diagnostic imaging

## Abstract

**Background:**

Multimodal large language models (MLLMs) have emerging potential for interpreting medical images and text, but their performance in orthopedic imaging tasks and the influence of prompt configuration remain insufficiently studied.

**Objective:**

This study aimed to evaluate the performance of commercial and open-source MLLMs for diagnosing and staging osteonecrosis of the femoral head (ONFH) and to assess how different prompt configurations affect model performance.

**Methods:**

This single-center retrospective diagnostic accuracy study included 159 radiograph patients contributing 318 hip-level observations and 170 magnetic resonance imaging (MRI) patients contributing 340 hip-level observations; 55 patients with both modalities formed the multi-image (MI) subgroup between July 2023 and December 2024. Four MLLMs were evaluated: GPT-4o, Claude 3.7 Sonnet, Qwen2.5-VL 72B, and Gemma 3 27B. Three prompt configurations were tested: single image (SI), image plus radiology description (ID), and MI. Model performance was assessed for ONFH detection; early- versus late-stage differentiation; detailed grading using the Ficat, Association Research Circulation Osseous (ARCO), and Steinberg systems; and grading reliability using intraclass correlation coefficients (ICCs).

**Results:**

Model performance varied by prompt configuration and imaging input. For ONFH detection, the SI configuration yielded a mean detection area under the receiver operating characteristic curve (AUC) of 0.55 (SD 0.03), whereas the ID configuration achieved a mean detection AUC of 0.91 (SD 0.01). In radiograph-based ONFH detection, ID input achieved a mean accuracy of 0.88 (SD 0.01); in MRI-based ONFH detection, ID input achieved a mean accuracy of 0.85 (SD 0.01). For early- versus late-stage differentiation, the mean accuracy was 0.65 (SD 0.11) with SI input, 0.78 (SD 0.04) with ID input, and approximately 0.59 (SD 0.10) with MI input. For detailed grading, ID input improved mean accuracy across the Ficat, ARCO, and Steinberg systems compared with SI input. In ARCO grading reliability analysis, the mean MLLM ICC was 0.51 (SD 0.18) for SI, 0.97 (SD 0.02) for ID, and 0.49 (SD 0.15) for MI; surgeon interrater and intrarater ICCs were 0.70 and 0.81, respectively. In the commercial versus open-source model comparison, no significant overall difference was observed between model groups (*P*=.83).

**Conclusions:**

Prompt configuration strongly influenced MLLM performance in ONFH diagnosis and staging. Pairing images with deidentified radiology descriptions improved diagnostic and grading performance, whereas MI input did not provide consistent additional benefit in this retrospective single-center evaluation. These findings support the potential role of MLLMs as assistive tools in human-AI orthopedic imaging workflows, but external validation, careful input standardization, and prospective clinical evaluation are needed before clinical deployment.

## Introduction

Osteonecrosis of the femoral head (ONFH) is a common progressive musculoskeletal condition primarily affecting young and middle-aged adults [1,2], with over 20,000 new cases annually in the United States [3]. Early ONFH is often asymptomatic and bilateral; without timely intervention, over half of affected hips may collapse and require total hip arthroplasty (THA) within a few years [4,5]. Given that THA in young patients is associated with higher revision rates and substantial long-term health care costs, accurate early diagnosis and precise staging are critical for guiding joint-preserving treatments, delaying disease progression, and improving long-term functional outcomes.

Radiographic assessment remains the cornerstone of ONFH diagnosis, but standard X-rays lack sensitivity in early disease [6]. Magnetic resonance imaging (MRI) improves early detection but provides limited assessment of late-stage collapse. Widely used grading systems, such as Ficat, Association Research Circulation Osseous (ARCO), and Steinberg, help standardize evaluation [7], but they differ in criteria and are subject to considerable interobserver variability [8,9]. These limitations highlight the need for diagnostic tools that can integrate multimodal information and enhance consistency across readers.

AI is a promising tool, with multimodal large language models (MLLMs) capable of processing both imaging and textual data, recently emerged as powerful tools in medical imaging [10-13]. Unlike traditional deep learning (DL) models trained for single diagnosis tasks [14-16], MLLMs can integrate imaging features with clinical context, perform higher-order reasoning, and generate structured reports [17-19]. These capabilities position MLLMs as potential assistants for diagnostic interpretation in complex clinical workflows.

Despite their promise, MLLM applications in clinical practice still face challenges [20]. In particular, limited translational research from computational models to clinical applications constrains the practical implementation of these advanced technologies. Prior studies have largely focused on testing commercial models and single-image tasks [10,11,21]. Commercial systems face challenges in cost, accessibility, and adaptability, whereas open-source models may offer more feasible alternatives for clinical adoption. To date, no systematic study has compared commercial and open-source MLLMs in complex orthopedic diagnostic tasks. In addition, the exploration of prompt engineering design for diagnosis has been limited—particularly in combining imaging with clinical text or integrating multimodal inputs. It remains unclear whether optimized prompting strategies can enhance model performance and increase clinical use. These gaps underscore the need to test the full clinical workflow.

To address these gaps, we systematically explored the potential of MLLMs in clinical applications. We evaluated 4 advanced models—2 commercial (ChatGPT-4o, Claude 3.7) and 2 open-source (Qwen2.5-VL, Gemma-3)—on core ONFH-related tasks: diagnosis, early- or late-stage differentiation, and staging. Furthermore, we examined the effect of prompt configuration by comparing single image (SI), image plus radiology description (ID), and multi-image (MI) inputs across different grading systems with quantitative metrics.

Therefore, the objective of this study is to evaluate, from a clinical observation perspective, whether MLLMs can serve as an assistive tool for diagnosing and staging ONFH within a human-machine collaborative workflow. Specifically, we aim to assess (1) whether MLLMs can diagnose and stage ONFH across commonly used classification systems, (2) whether different models (particularly open-source models) exhibit performance variations in clinical diagnosis and staging, and (3) whether prompting designs for different clinical contexts can enhance model performance and reliability.

## Methods

### Ethical Considerations

This retrospective study was reviewed and approved by the Ethics Committee of the Second Affiliated Hospital and Yuying Children’s Hospital of Wenzhou Medical University (approval number 2025-K-282‐01; approval date: February 8, 2025). The approval was obtained before any study-specific research procedures were initiated, including data extraction for this study, data deidentification, MLLM evaluation, and statistical analysis. Eligible cases were retrospectively identified from historical imaging data generated during routine clinical care between July 2023 and December 2024. Because this study involved only retrospective analysis of deidentified clinical imaging data and did not involve active patient intervention, the requirement for written informed consent was waived by the ethics committee. All personal identifiers were removed before analysis, and all data were handled in accordance with institutional privacy and confidentiality requirements. The requirement for written informed consent was waived by the Ethics Committee because this retrospective study used deidentified clinical imaging data and involved no active patient intervention.

### Data Collection and Preparation

This was a single-center retrospective diagnostic accuracy study conducted at the Second Affiliated Hospital and Yuying Children’s Hospital of Wenzhou Medical University, a tertiary referral hospital in Wenzhou, China. Potentially eligible cases were systematically identified from the in-house INFINITY picture archiving and communication system (PACS). The search included patients who underwent standardized anteroposterior pelvic radiographs or multisequence pelvic MRI for suspected or confirmed ONFH between July 2023 and December 2024. MRI examinations included at least T1-weighted imaging (T1WI) and T2-weighted fat-suppressed (T2FS) sequences.

Cases were screened in a stepwise manner. First, imaging records during the study period were retrieved from the PACS according to modality and clinical indication. Second, eligibility was assessed based on the predefined inclusion and exclusion criteria listed in Table S1 in Multimedia Appendix 1. Third, 2 senior radiologists reviewed image quality and diagnostic relevance (one with 15 years of experience and the other with 17 years of experience). Cases with incomplete imaging, severe artifacts, insufficient field of view, prior hip arthroplasty, or other findings that precluded reliable ONFH grading were excluded. For eligible MRI cases, 2 radiologists jointly selected representative slices for each patient, prioritizing images showing (1) maximal lesion diameter, (2) key diagnostic features, and (3) optimal lesion-to-marrow contrast. All eligible cases meeting the criteria during the study period were included consecutively; no random sampling was performed.

This single-center retrospective diagnostic accuracy study included 159 radiographic patients contributing 318 hip-level observations and 170 MRI patients contributing 340 hip-level observations; 55 patients with both modalities formed the MI subgroup. The distributions by disease status and laterality are detailed as follows: radiographs (18 non-ONFH, 141 ONFH [98 unilateral, 43 bilateral]) and MRI (24 non-ONFH, 146 ONFH [84 unilateral, 62 bilateral]).

### Imaging Grading Systems

To test the model’s generalization ability, 3 established imaging grading systems were selected for ONFH classification: the Ficat system [22], the ARCO system [23], and the Steinberg system [24]. The specific criteria for each system are detailed in Table S2 in Multimedia Appendix 1. Briefly, the Ficat system primarily emphasizes radiographic changes and femoral head collapse, the ARCO system incorporates radiographic and MRI findings with subcategorization by femoral head depression, and the Steinberg system provides a more quantitative severity framework based on lesion extent and structural progression. ARCO staging was operationalized using the radiographic and MRI criteria available in this study. Bone scintigraphy was not performed or used for reference-standard assignment. In the radiograph-only cohort, no hip was assigned to ARCO stage 1; radiographically normal hips were classified as ARCO stage 0. This operational category indicated the absence of radiographic abnormalities and did not correspond to asymptomatic ARCO stage 0 in the published 2019 ARCO criteria. In the MRI cohort, ARCO stage 1 was assigned when radiographs were normal, but MRI showed findings compatible with ONFH, such as a band-like low-signal-intensity lesion around the necrotic area. Because bone scintigraphy was unavailable for most patients, the ARCO results should be interpreted as radiograph or MRI-based operationalized ARCO staging rather than strict application of the complete 2019 ARCO staging system.

### Surgeon Grading and Reference Standard Establishment

Two senior orthopedic surgeons (A: 18 years of experience; B: 15 years of experience) and 1 junior orthopedic surgeon (C: 5 years of experience) independently graded all images using the Ficat, ARCO, and Steinberg systems. Their results were used for comparison with MLLM performance.

The reference standard was established independently at the hip level by a panel of another 4 experts, comprising 2 musculoskeletal radiologists and 2 additional senior orthopedic surgeons (average of 20 years of experience). Each panel member independently evaluated every hip using the prespecified diagnostic and staging criteria and was blinded to the MLLM outputs and the assessments of the comparison surgeons. Individual assessments were recorded before the consensus discussion. The mean interrater intraclass correlation coefficient (ICC) across the 3 grading systems was 0.84 (SD 0.05). Disagreements were resolved through panel discussion, and the final consensus diagnosis and stage for each hip served as the reference standard.

### Selection of MLLMs

Model selection was based on availability and reported multimodal capabilities when model evaluation began in April 2025. GPT-4o (released May 13, 2024) and Claude 3.7 Sonnet (released February 24, 2025) were selected as representative leading commercial models with strong reported image understanding and multimodal reasoning capabilities. Qwen2.5-VL-72B-Instruct (released January 26, 2025) and Gemma 3 27B instruction-tuned (released March 12, 2025) were selected as recently released, high-capacity open-source vision-language models. The largest available variants of these open-source model families were selected to maximize their potential visual reasoning capabilities.

Model evaluations began in April 2025 and were conducted using GPT-4o (model ID: gpt-4o-2024-11-20), Claude 3.7 Sonnet (model ID: claude-3‐7-sonnet-20250219), Qwen2.5-VL-72B-Instruct (model ID: qwen2.5-vl-72b-instruct), and Gemma 3 27B instruction-tuned (checkpoint/model ID: google/gemma-3-27b-it). All 4 models were evaluated during the same study period using standardized prompts and image inputs.

### Prompt Design

**Figure 1.**
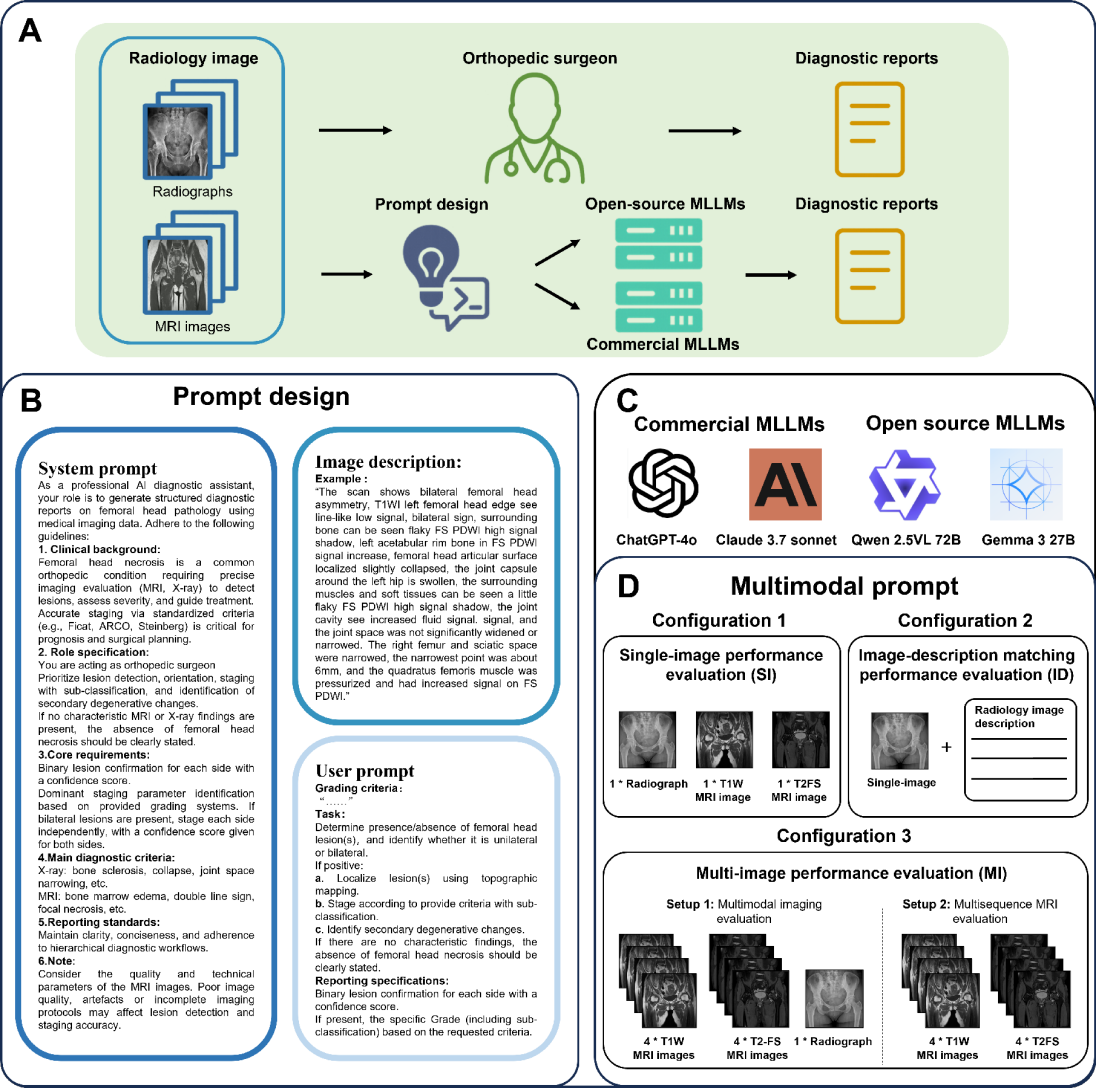
Study workflow and multimodal prompt configurations. (A) Multimodal large language model (MLLM) testing workflow. (B) Grading prompt design framework and image description example. (C) Commercial and open-source MLLMs evaluated. (D) Multimodal prompt configurations: single image (SI), image with description (ID), and multiple images (MI). MI included setup 1 (X-ray + magnetic resonance imaging [MRI] slices) and setup 2 (MRI slices only). MIS1: multi-image setup 1; MIS2: multi-image setup 2; T1WI: T1-weighted imaging; T2FS: T2-weighted fat-suppressed sequences.

To address diagnostic refusal issues and complete clinical tasks in a standardized format, we developed a systematic prompt strategy (Figure 1B), including a system prompt and a user prompt. A detailed prompt framework and representative radiology description examples are shown in Table S3 in Multimedia Appendix 1.

### Multimodal Prompt Configuration Design for MLLMs

To investigate whether different multimodal prompt configurations impact performance and enhance clinical utility, we evaluated 2 strategies in prompt engineering. Three multimodal prompt configurations (Figure 1D) were tested to assess MLLM performance across input types:

1. SI: This configuration establishes a baseline for the model’s zero-shot visual inference capabilities and tests the model’s ability to diagnose and grade using a single medical image per patient. The tests included independent evaluations using one of the following inputs: (1) 1 anteroposterior pelvic radiograph, (2) 1 representative T1WI MRI slice, or (3) 1 representative T2FS MRI slice. All 3 grading systems were used.
2. ID: Simulating the workflow of clinical surgeons, the model is provided with images and corresponding image descriptions to verify whether multimodal text-image correspondence prompts can enhance model performance. Prior work suggests that combining images with corresponding textual descriptions can enhance cross-modal alignment and support multimodal reasoning in vision-language models [25-27]. The models received the same single images as the SI (X-ray, T1WI, or T2FS), accompanied by a corresponding “radiology image description” text. The radiology descriptions used in the ID configuration were extracted from the objective findings section of routine clinical radiology reports generated by on-duty reporting and supervising radiologists during daily clinical practice; they were not created specifically for this study. The radiologists who participated in image screening and reference-standard establishment did not author the clinical reports used for the ID prompt descriptions. During data extraction, patient identifiers, diagnostic names, staging information, and conclusion-based diagnostic statements were removed, leaving only descriptions of visible imaging findings. The reference standard was established independently, and the assessors were blinded to the final diagnostic and staging labels contained in the original clinical reports.
3. MI: This configuration evaluates the model’s multiview integration capabilities and simulates the real-world clinical requirement of synthesizing information across multiple images. We aimed to test whether MLLMs could correlate features across different images to improve grading accuracy, specifically using the ARCO system. For each case, all MI images were submitted simultaneously within a single model call using the same ARCO grading prompt. Images were organized in a fixed order for consistency, but no explicit fusion strategy or method was imposed. No additional investigator-defined maximum token limit was set beyond the default constraints of each model interface. Two setups were tested:
  - Multi-image setup 1 (MIS1): All 4 models were tested. The inputs included 1 anteroposterior pelvic radiograph followed by 4 T1WI MRI slices and 4 T2FS MRI slices from the same patient.
  - Multi-image setup 2 (MIS2): It evaluated models (Claude and Gemma) that performed well in the SI-MRI tests. The inputs included 4 representative T1WI MRI slices, followed by 4 T2FS MRI slices from the same patient.

To ensure comparability across models, all MLLMs were evaluated using the same case order, image inputs, prompt templates, task instructions, and output format requirements within each prompt configuration. All models received the same images, which were exported directly from the institutional PACS in JPG format. Apart from deidentification, no image enhancement, cropping, or other postprocessing was performed before model evaluation in order to approximate the image-input conditions of routine clinical practice.

We provided the surgeons with the same images and corresponding radiological descriptions used in the ID configuration to simulate routine clinical practice. Therefore, the comparison between surgeons and MLLMs under the ID configuration was based on matched input information. The SI configuration was designed to assess zero-shot image interpretation, whereas the MI configuration was designed to explore MI reasoning.

### Performance Evaluation

#### Diagnostic Performance of ONFH Detection

ONFH detection was evaluated based on each model’s ability to diagnose the presence or absence of ONFH, using confidence scores ranging from 0 to 1 for each hip. The primary metric was the area under the receiver operating characteristic curve (AUC). Binary classification metrics, including accuracy, sensitivity, specificity, precision, recall, and *F*_1_-score, were calculated using a prespecified confidence-score threshold of 0.5. Model outputs with confidence scores of 0.5 or higher were classified as positive for ONFH, whereas scores below 0.5 were classified as negative.

Accuracy = (TP + TN)/(TP + TN + FP + FN)

Sensitivity = TP/(TP + FN)

Specificity = TN/(TN + FP)

Where TP is true positive, TN is true negative, FP is false positive, and FN is false negative.

#### Performance of Early- or Late-Stage ONFH Differentiation

Performance in early versus late ONFH staging was assessed. Reference grades were categorized as “early” (Ficat 1‐2; ARCO 0‐2; Steinberg 0‐3) or “late” (Ficat 3‐4; ARCO 3‐4; Steinberg 4‐6). Precision, recall, *F*_1_-score, and overall accuracy were calculated from these categorical predictions; no additional probability threshold was applied.

Precision = TP/(TP + FP)

Recall = TP/(TP + FN)

*F*_1_-score = 2 × (precision × recall)/(precision + recall)

#### Detailed Grading Performance

Detailed grading performance, including subgrades, was assessed using the Ficat, ARCO, and Steinberg systems. Key metrics included overall grading accuracy (the proportion of grades matching the reference). Confusion matrices illustrated classification patterns and errors.

### Reliability Assessment

Grading reliability was assessed using ICCs. Surgeon interrater reliability was calculated from the independent grading results of three orthopedic surgeons. Surgeon intrarater reliability was assessed by asking the surgeons to regrade a randomized subset of images after a 2-week interval. MLLM output stability was assessed by comparing 2 independent runs on the same dataset for each input configuration (SI, ID, and MI). Reliability analysis was limited to ARCO grading because ARCO was the only grading framework that was consistently applied across all prompt configurations, imaging cohorts, MLLM models, and clinical readers, including the MI settings. This approach allowed for a comparable run-to-run repeatability assessment for MLLMs and agreement analysis with physician grading across the full study design. ICCs were calculated using a 2-way mixed-effects model with absolute agreement, where values >0.75 were excellent, 0.5 to 0.75 fair to good, and <0.5 poor, with significance at *P*<.05 [28].

### Statistical Analysis

Statistical analyses were performed using SPSS v26.0 (IBM Corp) and custom statistical scripts for clustered resampling analyses. The individual hip within each imaging modality was retained as the primary unit of analysis because disease status and stage may differ between sides. Statistical significance was defined as a 2-sided *P*<.05.

The sample size calculation was performed for the overall cohort and indicated that 288 hips were required, assuming an expected accuracy of 75%, a 95% confidence level, and a 5% margin of error. The final cohort included 658 modality-specific hip observations, comprising 318 radiographic and 340 MRI observations, exceeding the overall sample size requirement. No separate sample size calculations were performed for modality-specific subgroups or individual disease-stage categories.

Descriptive statistics were used to summarize cohort characteristics. To account for within-patient correlations arising from bilateral hips and from patients contributing observations to more than 1 imaging modality, generalized estimating equations with patient ID as the clustering variable were used for binary correctness outcomes. For AUC, sensitivity, specificity, *F*_1_-score, staging accuracy, and ICC, 95% CIs were estimated using patient-clustered bootstrap resampling with 2000 repetitions, in which patients rather than individual hip observations were resampled, and all hip-level observations from selected patients were retained. Paired patient-clustered bootstrap analysis was used to compare SI and ID configurations. A one-hip-per-patient sensitivity analysis was also conducted to assess robustness. When multiple pairwise comparisons were performed within the same analysis family, Bonferroni correction was applied to control for multiple testing.

## Results

### Baseline Cohort Characteristics

**Figure 2.**
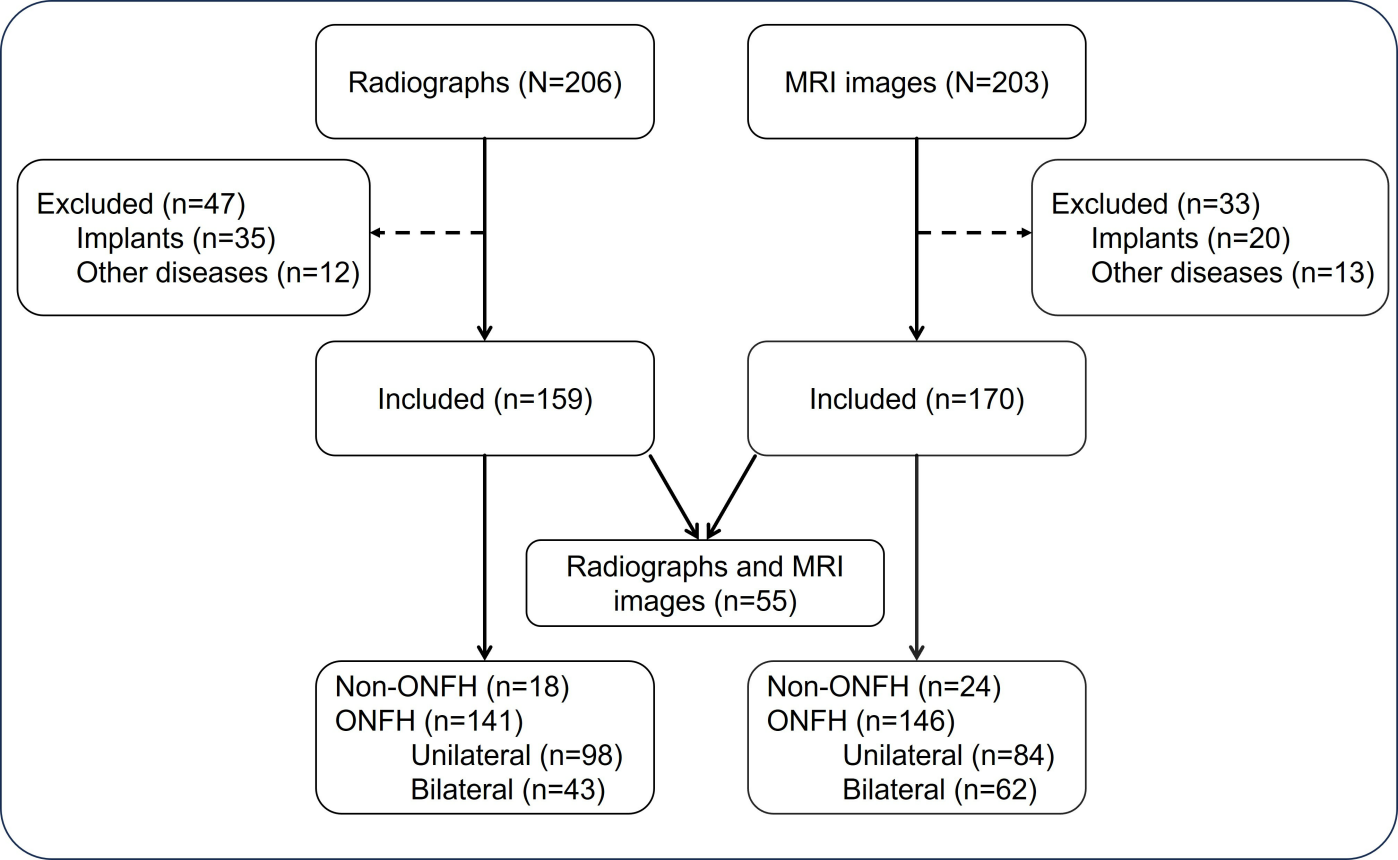
Data collection flowchart. MRI: magnetic resonance imaging; ONFH: osteonecrosis of the femoral head.

The patient selection and cohort construction process is shown in Figure 2. The patient cohort included 159 radiographic patients contributing 318 hip-level observations and 170 MRI patients contributing 340 hip-level observations; 55 patients with both modalities formed the MI subgroup (Figure 2). Radiographic patients (mean age 59.2, SD 14.5 y; 81 male patients, 78 female patients) had a higher proportion of late-stage ONFH (119/318 hips, 37%), while MRI patients (mean age 54.5, SD 17.2 y; 101 male patients, 69 female patients) had more early-stage disease (278/340 hips, 82%; Table 1).

**Table 1.** Distribution of osteonecrosis of the femoral head (ONFH) grades (per-hip) in the study cohort^a^.

Grade	Radiograph			MRI^b^ image		
	Ficat, n (%)	ARCO^c^, n (%)	Steinberg, n (%)	Ficat, n (%)	ARCO, n (%)	Steinberg, n (%)
0	—^d^	135 (42.6)	125 (39.3)	—	91 (26.8)	120 (35.3)
1	135 (42.6)	0 (0.0)	3 (0.9)	133 (39.1)	61 (17.9)	39 (11.5)
2	76 (23.9)	63 (19.8)	68 (21.4)	145 (42.6)	109 (32.1)	100 (29.4)
3	37 (11.6)	43 (13.5)	4 (1.3)	26 (7.6)	45 (13.2)	16 (4.7)
4	70 (22.0)	76 (24.0)	41 (12.9)	36 (10.6)	34 (10)	34 (10)
5	—	—	46 (14.5)	—	—	22 (6.5)
6	—	—	31 (9.7)	—	—	9 (2.6)

a Distribution of osteonecrosis of the femoral head grades according to the Ficat, Association Research Circulation Osseous, and Steinberg systems for the radiographic and magnetic resonance imaging cohorts.

b MRI: magnetic resonance imaging.

c ARCO: Association Research Circulation Osseous.

d Not applicable.

### Diagnostic Performance of ONFH Detection

**Figure 3.**
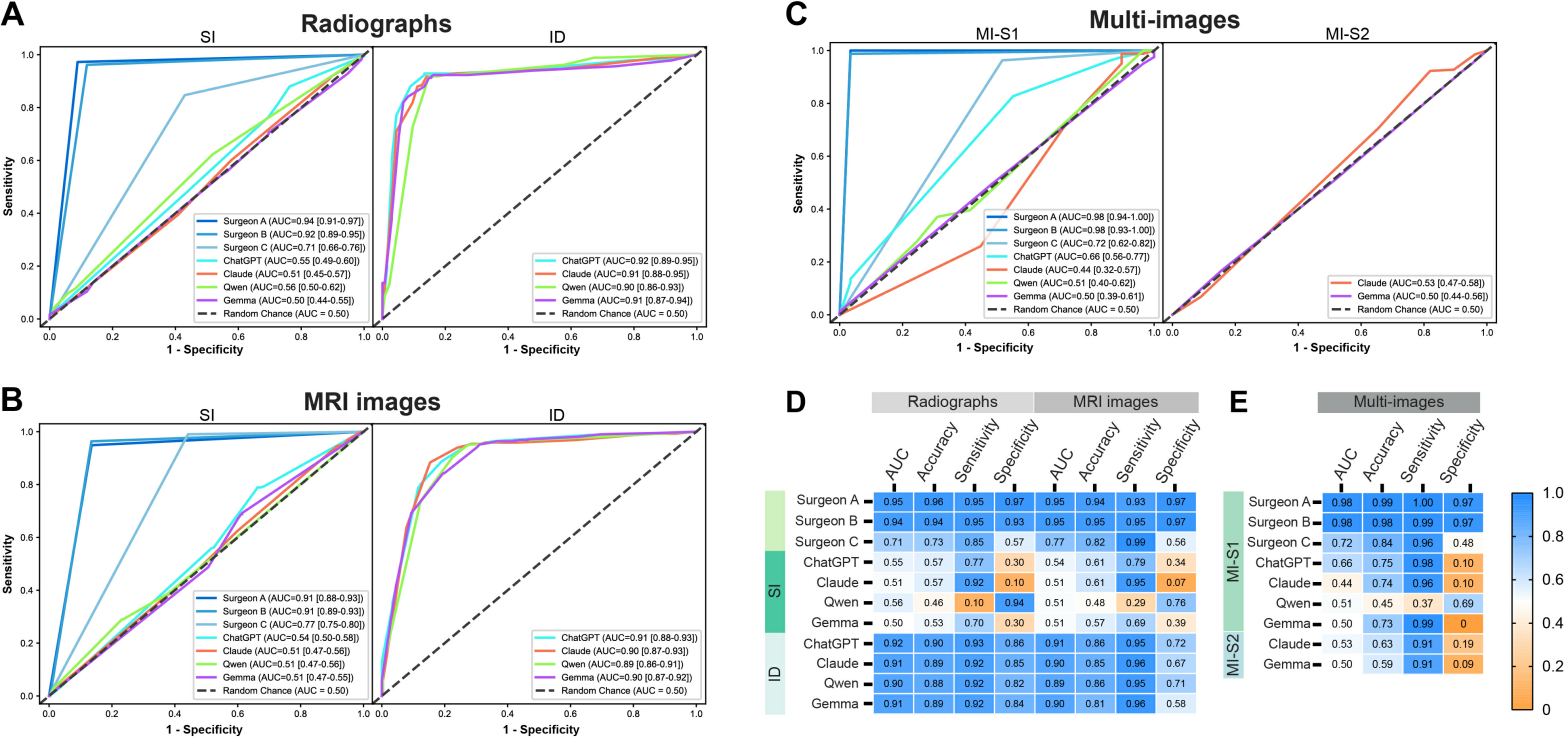
Diagnostic performance based on receiver operating characteristic (ROC) curves and binary classification metrics. (A) ROC curves for surgeons and multimodal large language models (MLLMs) with radiographic inputs (single image [SI], image plus radiology description [ID]). (B) ROC curves for surgeons and MLLMs with magnetic resonance imaging (MRI) inputs (SI, ID). (C) ROC curves for surgeons and MLLMs in the multi-image (MI) configuration (multi-image setup 1 [MIS1], multi-image setup 2 [MIS2]). (D) Performance metrics at a 0.5 threshold. AUC: area under the receiver operating characteristic curve.

ONFH diagnosis by MLLMs strongly depended on prompt configuration (Figure 3). Surgeons showed high diagnostic accuracy, correlating with experience (A>B>C; *P*<.001). With SI input, MLLMs showed limited diagnostic discrimination, with AUCs ranging from 0.50 to 0.58 and a mean AUC of 0.55 (SD 0.03). ID input substantially improved detection performance, with AUCs ranging from 0.90 to 0.93 and a mean AUC of 0.91 (SD 0.01). Patient-clustered bootstrap analyses showed that ID significantly increased detection AUC compared with SI for all 4 models in both radiograph and MRI cohorts (all *P*<.001). Generalized estimating equation (GEE) analyses similarly showed significantly higher diagnostic correctness with ID than with SI for every model in both imaging cohorts (all *P*<.001). In contrast, MI input showed limited and inconsistent diagnostic discrimination, with a mean AUC of 0.53 (SD 0.08). The one-hip-per-patient sensitivity analysis yielded similar results, with AUCs of 0.52 (95% CI 0.50‐0.54) for SI, 0.92 (95% CI 0.91‐0.93) for ID, and 0.55 (95% CI 0.47‐0.63) for MI. Model-specific AUC estimates and 95% CIs are provided in Multimedia Appendix 2.

### Performance of Early- or Late-Stage ONFH Differentiation

**Figure 4.**
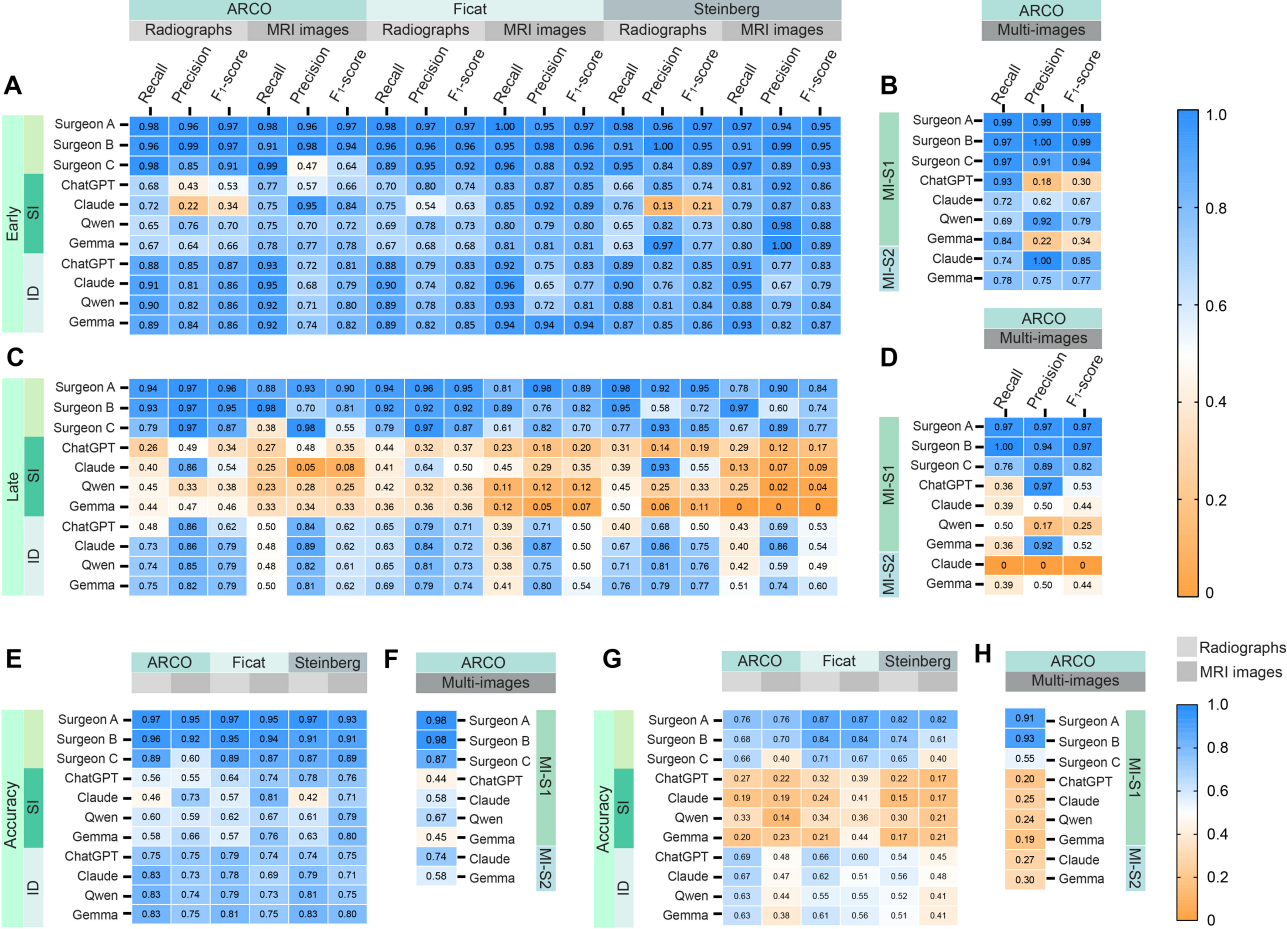
Early- and late-stage osteonecrosis of the femoral head (ONFH) differentiation and detailed grading accuracy. (A) Performance metrics (precision, recall, *F*_1_-score) for early ONFH differentiation (single image [SI], image plus radiology description [ID]). (B) Performance metrics for early ONFH differentiation (multi-image setup 1 [MIS1], multi-image setup 2 [MIS2]). (C) Performance metrics for late ONFH differentiation (SI, ID). (D) Performance metrics for late ONFH differentiation (MIS1, MIS2). (E) Accuracy of early- and late-stage ONFH differentiation (SI, ID). (F) Accuracy for early and late differentiation (MIS1, MIS2). (G) The detailed accuracy of surgeons and multimodal large language models (MLLMs) in grading ONFH differentiation in the SI and ID configurations. (H) The detailed accuracy of surgeons and MLLMs in grading ONFH differentiation in the MIS1 and MIS2 configurations. ARCO: Association Research Circulation Osseous; MI: multi-image; MRI: magnetic resonance imaging.

For early- and late-stage ONFH differentiation, MLLM performance was also strongly influenced by prompt configuration, with early-stage detection being better than late-stage detection (Figure 4). Across the Ficat, ARCO, and Steinberg systems, the mean early- or late-stage accuracy increased from approximately 0.65 (SD 0.11) with SI input to 0.78 (SD 0.04) with ID input. For ARCO-based early or late differentiation, ID significantly improved accuracy over SI for all 4 models in the radiograph cohort (all *P*<.001). In the MRI cohort, ID significantly improved ARCO early or late accuracy for Qwen and ChatGPT (both *P*<.001) and Gemma (*P*=.01), whereas no significant improvement was observed for Claude (*P*=.50). MI input did not show a consistent advantage, with the mean ARCO early or late accuracy of approximately 0.59 (SD 0.10).

### Detailed Grading Performance

Detailed grading performance remained more challenging than binary ONFH detection. Surgeons achieved a mean accuracy of 0.71 (SD 0.19). Nevertheless, ID input consistently improved grading accuracy compared with SI input across the 3 grading systems (Figure 4G,H). The mean detailed grading accuracy increased from 0.34 (SD 0.08) to 0.59 (SD 0.04) for Ficat, from 0.22 (SD 0.05) to 0.56 (SD 0.11) for ARCO, and from 0.19 (SD 0.05) to 0.49 (SD 0.05) for Steinberg. Patient-clustered bootstrap analyses showed that ID significantly outperformed SI for all models in both radiograph and MRI cohorts (*P*<.001). MI input provided limited detailed ARCO grading performance, with a mean accuracy of approximately 0.24 (SD 0.03).

### Performance Comparison of Different MLLMs

**Figure 5.**
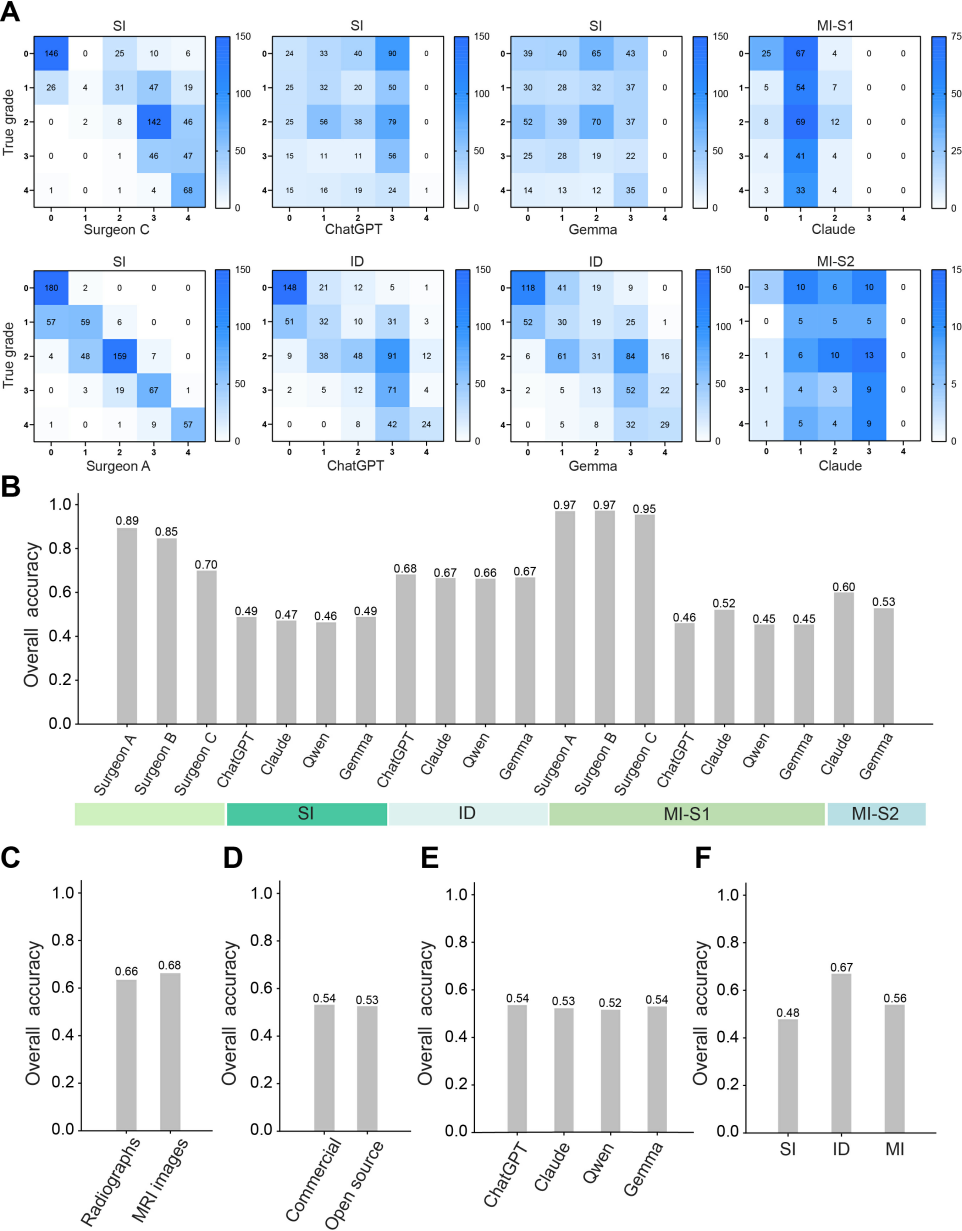
Overall multimodal large language model (MLLM) performance analysis. (A) Confusion matrix of surgeons, ChatGPT, and Qwen in the Association Research Circulation Osseous (ARCO) grading system under the magnetic resonance imaging (MRI) single image (SI) and image plus radiology description (ID) configurations. (B) Overall accuracy for surgeons and MLLMs across the SI, ID, multi-image setup 1 (MIS1), and multi-image setup 2 (MIS2) configurations. (C) Comparison of overall accuracy between the radiographs and MRI images. (D) Comparison of overall accuracy between commercial and open-source MLLMs. (E) Overall accuracy of individual MLLMs. (F) Comparison of overall accuracy across multimodal prompt configurations. Bar plots show descriptive overall accuracy across models, imaging inputs, and prompt configurations. Formal patient-clustered statistical comparisons between SI, ID, and MI configurations are provided in Multimedia Appendix 2.

This study compared the performance of different MLLMs, distinguishing between commercial and open-source solutions. Consistent with the bootstrap analysis, no significant overall difference was found between commercial and open-source models, though some model-specific variations existed (Figure 5C,D). Furthermore, individual models exhibited notable heterogeneity in diagnostic strategies for ONFH detection. Claude and Gemma demonstrated high sensitivity (mean 0.81, SD 0.13) but low specificity (mean 0.30, SD 0.13), suggesting screening use, while Qwen adopted a high-specificity (mean 0.85, SD 0.14), low-sensitivity (mean 0.20, SD 0.11) approach suited for confirmatory roles; ChatGPT showed a balanced sensitivity and specificity. This model-specific behavior was modality-contingent, with open-source Gemma showing higher accuracy than commercial ChatGPT in MRI-based differentiation, but ChatGPT showing higher accuracy with radiographs (Figure 5C,E).

### Impact of Multimodal Prompt Configurations

Prompt configuration had a marked effect on model performance (Figure 5). Across all models and imaging modalities, the mean detection AUC increased from 0.55 (SD 0.03) with SI input to 0.91 (SD 0.01) with ID input, corresponding to an average AUC gain of 0.37 (SD 0.03). The AUC improvement was significant for each model in both radiograph and MRI cohorts after patient-clustered bootstrap correction (all *P*<.001). ID input also improved ARCO detailed grading accuracy from 0.22 to 0.56 and ARCO early- or late-stage accuracy from 0.59 to 0.79. By contrast, MI input did not produce consistent performance gains over SI input. In paired patient-clustered bootstrap comparisons, MI AUCs were similar to SI AUCs, with mean AUC differences of −0.03 (SD 0.07) for MIS1 versus radiograph SI, −0.05 (SD 0.10)for MIS1 versus MRI SI, and −0.002 (SD 0.03) for MIS2 versus MRI SI; most model- and modality-specific comparisons were not significant. In contrast, MI AUCs were significantly lower than the corresponding ID AUCs, with mean AUC differences of −0.38 (SD 0.09) for MIS1 versus radiograph ID, −0.40 (SD 0.10) for MIS1 versus MRI ID, and −0.37 (SD 0.05) for MIS2 versus MRI ID across all comparisons (all *P*<.001). These findings suggest that simply increasing the number of images did not reliably improve current MLLM reasoning in this task. Detailed paired bootstrap and GEE results are provided in Multimedia Appendix 2.

### Reliability of Grading

To assess grading reliability, we evaluated ARCO grading consistency using ICCs (Tables 2 and 3). Reliability analysis was limited to ARCO because it was the only grading framework applied across all prompt configurations, including MI. Human readers showed moderate-to-good interrater agreement, with ICCs of 0.74 for radiographs and 0.72 for MRI images. In the SI configuration, MLLM repeatability varied substantially across models and modalities, with a mean ICC of 0.51 (SD 0.18). ID input markedly improved model repeatability, with a mean ICC of 0.97 (SD 0.02) and consistently high ICCs across models. In contrast, MI configurations showed lower and less stable reliability, with mean ICCs of 0.52 (SD 0.18) for MIS1 and 0.43 (SD 0.01) for MIS2.

**Table 2.** Intraclass correlation coefficients for multimodal large language model Association Research Circulation Osseous grading reliability^a^.

Imaging input	Prompt configuration	ChatGPT	Claude	Qwen	Gemma
Radiographs	SI^b^	0.76	0.68	0.52	0.29
Radiographs	ID^c^	0.97	0.98	0.96	0.97
MRI^d^ images	SI	0.38	0.53	0.37	0.44
MRI images	ID	0.95	0.99	0.96	0.93
Multi-images	MIS1^e^	0.37	0.75	0.37	0.59
Multi-images	MIS2^f^	—^g^	0.43	—	0.45

a Intraclass correlation coefficients indicate intramodel repeatability for Association Research Circulation Osseous grading across two independent runs.

b SI: single image.

c ID: image plus radiology description.

d MRI: magnetic resonance imaging.

e MIS1: multi-image setup 1.

f MIS2: multi-image setup 2.

g The em dash indicates that the corresponding model-configuration combination was not evaluated.

**Table 3.** Intraclass correlation coefficients for surgeon Association Research Circulation Osseous grading reliability^a^.

Reference surgeon	Imaging input	Surgeon A	Surgeon B	Surgeon C
Surgeon A	Radiographs	0.92	0.87	0.69
Surgeon A	MRI^b^ images	0.81	0.79	0.73
Surgeon B	Radiographs	—^c^	0.87	0.67
Surgeon B	MRI images	—	0.89	0.61
Surgeon C	Radiographs	—	—	0.72
Surgeon C	MRI images	—	—	0.78

a Intraclass correlation coefficients indicate surgeon Association Research Circulation Osseous grading reliability. Diagonal values indicate intrareader reliability, and off-diagonal values indicate pairwise interreader reliability.

b MRI: magnetic resonance imaging.

c The em dash indicates duplicate pairwise comparisons that are not repeated in the table.

To better characterize run-to-run variability, we additionally calculated exact agreement, within-one-stage agreement, and mean absolute ARCO stage difference. ID input showed the highest stability, with exact agreement of 0.79‐0.96 (mean 0.90, SD 0.05), within-one-stage agreement of 0.98‐1.00 (mean 0.99, SD 0.01), and mean absolute differences of 0.05‐0.23 stages (mean 0.11, SD 0.06). In contrast, SI input showed lower stability, with exact agreement of 0.44‐0.81 (mean 0.65, SD 0.16) and mean absolute differences of 0.22‐1.09 stages (mean 0.60, SD 0.27). MI configurations showed inconsistent repeatability, with exact agreement of 0.47‐0.85 (mean 0.67, SD 0.14) and mean absolute differences of 0.18‐0.74 stages (mean 0.44, SD 0.23).

## Discussion

### Principal Findings

This study evaluated whether MLLMs can assist in ONFH diagnosis and staging, whether performance differs across commercial and open-source models, and whether prompt configuration affects diagnostic reliability. The main findings were that prompt configuration strongly influenced MLLM performance, ID input substantially improved diagnostic and grading performance, MI input did not provide consistent additional benefit, and different models showed task-specific strengths without a consistent advantage of commercial over open-source models.

### Interpretation and Comparison With Previous Work

These findings should be interpreted in the context of previous work on medical imaging AI and multimodal models. In terms of diagnostic and staging performance, the evaluated MLLMs demonstrated diagnostic performance competitive with existing task-specific DL models in the ID configuration (Table S4 in Multimedia Appendix 1). For example, while prior studies reported an AUC of 0.80 for stage differentiation [29] and 88% accuracy for diagnosis [15] using DL, MLLMs in the ID configuration achieved a mean early/late classification accuracy of approximately 0.78 (SD 0.04) and a mean detection AUC of 0.91 (SD 0.01) for ONFH diagnosis. However, these values were obtained from different datasets, patient populations, study designs, and evaluation procedures and should not be interpreted as a direct performance comparison. By contrast, the SI configuration represented zero-shot image-only interpretation and showed substantially lower performance, underscoring the current limitations of autonomous MLLM-based diagnosis. Unlike task-specific DL models trained on labeled datasets, the MLLMs in this study were evaluated without task-specific training or fine-tuning. The comparison presented here serves only as a reference for diagnostic performance. These findings demonstrate the potential of MLLMs to support human-AI collaborative workflows in orthopedics. Traditional DL models are typically trained for single-label tasks, which limits their adaptability. In contrast, MLLMs can integrate both image and textual inputs without task-specific fine-tuning, enabling them to generalize across different grading systems and perform multiple task types. This multimodal capability and strong generalization ability demonstrate their promise for broader clinical application translation.

Most prior research has focused on commercial models [21,30-32] without systematically comparing the performance of different MLLMs in clinical tasks. However, this research has found that different models displayed modality-specific strengths (eg, Gemma > ChatGPT in MRI; Qwen > Claude in radiographs) and nuanced diagnostic strategies (eg, Qwen: high specificity for confirmation; Claude/Gemma: high sensitivity for screening). These findings have rarely been mentioned in previous comparative studies [17,19,33]. These observations suggest that different MLLMs may exhibit distinct performance tendencies under specific imaging conditions. Models with higher sensitivity may be more suitable for screening, where missed early ONFH could delay joint-preserving treatment, whereas models with higher specificity may be more appropriate for confirmatory support to reduce unnecessary examinations caused by false-positive results. Nevertheless, none of the evaluated models should currently be used independently for clinical decision-making.

From a clinical perspective, these results suggest that different MLLMs may be suited to different assistive roles rather than a single universal diagnostic use case. Furthermore, our research, along with other recent studies, indicates that open-source alternatives hold significant potential [34]. From a deployment perspective, commercial models offer easy access via cloud-based APIs but may involve ongoing costs, network latency, vendor dependence, and privacy, security, and data-governance issues. In contrast, open-source models support on-premises deployment, institutional customization, and version control and can better protect sensitive clinical data [35]. As this study focused on diagnostic performance, inference time, computational costs, and deployment requirements were not systematically measured; these aspects should be evaluated in future research.

The significant impact of multimodal prompt configuration design on model performance is well documented [36], and our study confirms that multimodal prompting enhances all MLLMs’ performance by radiology-description prompting. ID prompts, which combine images with radiologist-generated descriptions (extracted from original clinical reports without diagnostic conclusion information), significantly improve diagnostic accuracy and grading precision for both X-ray and MRI modalities. The accompanying radiology descriptions may improve performance by directing model attention toward clinically relevant imaging findings and facilitating cross-modal alignment [25-27]. Notably, this approach improves interrater agreement (ICC=0.96) to a level that approached or exceeded physician-reader agreement in selected comparisons [8,9]. Counterintuitively, the MI configuration, which mimics clinical workflows, failed to provide substantial benefits. Recent MI benchmarks have shown that although MLLMs perform well in many single-image tasks, they still have limitations in fine-grained perception, cross-image comparison, and MI reasoning [37]. Multi-image inputs may also increase the risk of context dilution and unstable attention allocation, where subtle ONFH findings on a key slice may be underweighted when multiple radiographs, T1WI, and T2FS images are submitted together. This explanation is consistent with recent work showing that hallucination and reasoning instability can increase in MI settings and may be influenced by interimage attention distribution [38]. The results suggest that integrating radiologist-generated image descriptions not only increases diagnostic performance but also enhances model stability. This suggests that the practical value of MLLMs may currently lie in integrating clinician-generated imaging descriptions with visual inputs within human-AI collaborative workflows, underscoring the practical value of human-AI collaboration in real-world clinical applications [39].

These findings suggest that MLLMs may have practical value as assistive tools within human-AI collaborative workflows in orthopedic imaging. When radiologist-generated imaging descriptions were available, MLLMs showed improved diagnostic and grading performance in selected tasks, supporting their potential role in standardizing ONFH assessment and organizing imaging findings into structured outputs. The comparable performance of open-source and commercial models also suggests that institutionally controlled and lower-cost deployment strategies may be feasible. However, these results should be interpreted as preliminary, and prospective multicenter studies are needed to evaluate workflow efficiency, clinical safety, and real-world use before implementation.

### Limitations

This study has several limitations. First, the single-center dataset limits generalizability. Differences across institutions in imaging protocols, disease prevalence, patient demographics, and annotation practices may affect model performance. External validation using multicenter datasets is therefore required before clinical application. Second, the MI analysis was exploratory; the MI cohort was relatively small, and we did not investigate the mechanisms underlying the underperformance of MI configurations. Third, the MRI subgroup contained a higher proportion of early-stage ONFH cases, and this class imbalance may have affected stage-specific performance estimates. Fourth, this study did not include a text-only control condition using radiology descriptions without images. Therefore, the relative contribution of textual radiology descriptions and image inputs to the improved ID performance could not be quantified. Fifth, repeatability was assessed mainly for ARCO grading, and although ICC and run-to-run variability metrics were reported, no established threshold currently defines clinically acceptable MLLM variability for ONFH staging. Moreover, deterministic generation settings could not be fully ensured across all models, which may have contributed to output variability. Finally, because bone scintigraphy was unavailable, ARCO stage 0 was operationally defined in this study as the absence of radiographic abnormalities, and ARCO-related results should therefore be interpreted within this radiograph- or MRI-based operational framework. Despite these limitations, our findings offer valuable insights into the potential of MLLMs in medical applications.

### Conclusions

This study highlights the importance of input design when applying MLLMs to medical imaging tasks. The broader implication is that MLLMs may be most useful not as stand-alone diagnostic systems but as assistive tools that help standardize imaging interpretation and integrate visual and textual clinical information within human-AI workflows. Open-source models may further support accessible and institutionally controlled deployment in musculoskeletal imaging. However, current MLLMs remain insufficient for independent diagnostic use, and future studies should focus on prospective validation, multicenter testing, robust MI reasoning, and workflow-level evaluation before clinical implementation.

## Supplementary material

10.2196/92919 Multimedia Appendix 1 Supplementary methodological tables, including patient inclusion and exclusion criteria, osteonecrosis of the femoral head grading criteria for the Ficat, Association Research Circulation Osseous, and Steinberg systems, prompt-engineering details, and a comparison of multimodal large language model performance with previously published deep learning models for osteonecrosis of the femoral head.

10.2196/92919 Multimedia Appendix 2 Supplementary performance and statistical analysis tables, including diagnostic performance, early or late staging performance, intraclass correlation coefficient–based reliability results, and statistical comparisons across imaging inputs, prompt configurations, multimodal large language models, and clinical readers.

## Abbreviations

ARCO: Association Research Circulation Osseous
AUC: area under the receiver operating characteristic curve
DL: deep learning
FN: false negative
FP: false positive
GEE: generalized estimating equation
ICC: intraclass correlation coefficient
ID: image plus radiology description
MI: multi-image
MIS1: multi-image setup 1
MIS2: multi-image setup 2
MLLM: multimodal large language model
MRI: magnetic resonance imaging
ONFH: osteonecrosis of the femoral head
PACS: picture archiving and communication system
SI: single image
T1WI: T1-weighted imaging
T2FS: T2-weighted fat-suppressed
THA: total hip arthroplasty
TN: true negative
TP: true positive
