# Supplementary material for: Prompt Configurations for Multimodal Large Language Models in Diagnosing and Staging Osteonecrosis of the Femoral Head: Multimodel Retrospective Observational Diagnostic Study
Source: J Med Internet Res. 2026 Aug 10;28:e92919. doi: 10.2196/92919 (PMC13455580; doi:10.2196/92919)
Supplement: Supplementary file 1 [file jmir-v28-e92919-s001.docx]

## Supplementary Table S1. Criteria for inclusion and exclusion of patients

| **Criteria** | **Content** |
| --- | --- |
| **Inclusion criteria** | (1) Patients aged ≥18 years who undergoing standardized AP pelvic imaging (X-ray or MRI) during the study period. This included both patients with suspected or confirmed ONFH and asymptomatic controls exhibiting normal hip imaging patterns. |
|  | (2) Adequate imaging quality, defined as clear visualization and localization of the femoral head, femoral neck, acetabulum, and joint space without significant rotational or flexion/extension artifacts. |
| **Exclusion criteria** | (1) Concomitant hip diseases affecting normal joint physiology (e.g., rheumatoid arthritis, ankylosing spondylitis, developmental hip dysplasia, previous hip preservation surgery, implants, or prostheses in the hip joint). |
|  | (2) Poor image quality, including incomplete anatomical coverage (e.g., >1/3 of the femoral head or acetabular rim missing) or severe motion artifacts obscuring relevant structures. |

Legend: Criteria for patient inclusion and exclusion.

Abbreviations: AP, anteroposterior pelvis; MRI, magnetic resonance imaging; ONFH, osteonecrosis of the femoral head.

## Supplementary Table S2. ONFH Grading criteria

| **Classification**  **system** | **Grade** | **Sub-**  **grade** | **Definition** |
| --- | --- | --- | --- |
| **Ficat** | 1 |  | Normal radiographs |
|  | 2 |  | Sclerotic or cystic lesions |
|  | 2 | 2A | No sign of subchondral collapse |
|  |  | 2B | Subchondral collapse (crescent sign on radiograph) without femoral head flattening |
|  | 3 |  | Femoral head flattening |
|  | 4 |  | Osteoarthritis with decreased joint space, articular collapse, or acetabular involvement |
|  |  |  |  |
| **ARCO** | 0 |  | All imaging normal |
|  | 1 |  | X-ray normal, MRI abnormal. A band lesion of low signal intensity around the necrotic area is seen on MRI. A cold spot is seen on bone scan. No changes are seen on plain radiographs. |
|  | 2 |  | X-ray abnormal, MRI abnormal. Osteosclerosis, focal osteoporosis or cystic changes are seen in the femoral head on plain radiographs or CT scan. Still there is no evidence of subchondral fracture, fracture in the necrotic portion or flattening of the femoral head. |
|  | 3 |  | Subchondral fracture on X-ray or CT. Subchondral fracture, fracture in the necrotic portion and/or flattening of the femoral head is seen on plain radiography or CT scan. |
|  |  | 3A | Femoral head depression ≤ 2 mm |
|  |  | 3B | Femoral head depression >2 mm |
|  | 4 |  | X-ray osteoarthritis. Osteoarthritis of the hip joint with joint space narrowing, acetabular changes and destruction are seen on plain radiographs. |
|  |  |  |  |
| **Steinberg** | 0 |  | All imaging normal |
|  | 1 |  | Normal findings on radiographs and abnormal MRI findings |
|  |  | 1A | Mild <15% of head affected |
|  |  | 1B | Moderate 15–30% |
|  |  | 1C | Severe: >30% |
|  | 2 |  | > 4 mm joint space; small osteophytes, slight sclerosis, or femoral condyle flattening |
|  |  | 2A | Mild <15% of head affected |
|  |  | 2B | Moderate 15–30% |
|  |  | 2C | Severe: >30% |
|  | 3 |  | Subchondral collapse and/or fracture |
|  |  | 3A | Mild: < 15% of articular surface |
|  |  | 3B | Moderate: 15–30% |
|  |  | 3C | Severe: > 30% |
|  | 4 |  | Femoral head flattening |
|  |  | 4A | Mild: <15% of surface and <2 mm depression |
|  |  | 4B | Moderate: 15–30% of surface or 2–4 mm depression |
|  |  | 4C | Severe: >30% of surface or >4 mm depression |
|  | 5 |  | Joint space narrowing with or without acetabular involvement |
|  | 6 |  | Advanced degenerative changes |

Legend: Criteria for the Ficat, ARCO, and Steinberg classification systems.

Abbreviations: ARCO, Association Research Circulation Osseous. MRI, magnetic resonance imaging. CT, computed tomography.

## Supplementary Table S3. Prompt Engineering

Prompt-grading

**System Prompt**

As a professional AI diagnostic assistant, your role is to generate structured diagnostic reports on femoral head pathology using medical imaging data. Adhere to the following guidelines:

1. **Clinical Background:**

Femoral head necrosis is a common orthopaedic condition requiring precise imaging evaluation (MRI, X-ray) to detect lesions, assess severity, and guide treatment.

Accurate staging via standardized criteria (e.g., Ficat, ARCO, Steinberg) is critical for prognosis and surgical planning.

2. **Role Specification:**

You are acting as orthopaedic surgeon

Prioritize lesion detection, orientation, staging with sub-classification, and identification of secondary degenerative changes. If no characteristic MRI or X-ray findings are present, the absence of femoral head necrosis should be clearly stated.

3. **Core Requirements:**

Binary lesion confirmation for each side with a confidence score.

Dominant staging parameter identification based on provided grading systems. If bilateral lesions are present, stage each side independently, with a confidence score given for both sides.

4. **Main Diagnostic Criteria:**

X-ray: bone sclerosis, collapse, joint space narrowing, etc.

MRI: bone marrow edema, double line sign, focal necrosis, etc.

5. **Reporting Standards:**

Maintain clarity, conciseness, and adherence to hierarchical diagnostic workflows.

6. **Note:**

Consider the quality and technical parameters of the MRI images. Poor image quality, artefacts or incomplete imaging protocols may affect lesion detection and staging accuracy.

**User Prompt-Ficat**

**GRADING CRITERIA：****Ficat**

1	Normal radiograph

2	Sclerotic or cystic lesions

2	2A	No sign of subchondral collapse

2B	Subchondral collapse (crescent sign on radiograph) without femoral head flattening

3	Femoral head flattening

4	Osteoarthritis with decreased joint space, articular collapse, or acetabular involvement

**TASK：**

Determine presence/absence of femoral head lesion(s)，and identify whether it is unilateral or bilateral.

If positive:

a. Localize lesion(s) using topographic mapping.

b. Stage according to provide criteria with sub-classification.

c. Identify secondary degenerative changes.

If there are no characteristic findings, the absence of femoral head necrosis should be clearly stated.

**REPORTING SPECIFICATIONS:**

Binary lesion confirmation for each side with a confidence score.

If present, the specific Grade (including sub-classification) based on the requested criteria.

**User Prompt-ARCO**

**GRADING CRITERIA：ARCO 2019 revised version**

0	All imaging normal

1	X-ray normal, MRI abnormal. A band lesion of low signal intensity around the necrotic area is seen on MRI. A cold spot is seen on bone scan. No changes are seen on plain radiographs.

2	X-ray abnormal, MRI abnormal. Osteosclerosis, focal osteoporosis or cystic changes are seen in the femoral head on plain radiographs or CT scan. Still there is no evidence of subchondral fracture, fracture in the necrotic portion or flattening of the femoral head.

3	Subchondral fractures on X-ray or CT. Subchondral fracture, fracture in the necrotic portion and/or flattening of the femoral head is seen on plain radiography or CT scan.

3A	Femoral head depression ≤ 2 mm

3B	Femoral head depression >2 mm

4	X-ray osteoarthritis. Osteoarthritis of the hip joint with joint space narrowing, acetabular changes and destruction are seen on plain radiographs.

**TASK：**

Determine presence/absence of femoral head lesion(s)，and identify whether it is unilateral or bilateral.

If positive:

a. Localize lesion(s) using topographic mapping.

b. Stage according to provide criteria with sub-classification.

c. Identify secondary degenerative changes.

If there are no characteristic findings, the absence of femoral head necrosis should be clearly stated.

**REPORTING SPECIFICATIONS:**

Binary lesion confirmation for each side with a confidence score.

If present, the specific Grade (including sub-classification) based on the requested criteria.

**User Prompt-Steinberg**

**GRADING CRITERIA：Steinberg classification**

0	All imaging normal

1	Normal finding on radiographs and abnormal MRI findings

1A	Mild <15% of head affected

1B	Moderate 15–30%

1C	Severe: >30%

2	Lucent and sclerotic changes in femoral head

2A	Mild: <15%

2B	Moderate 15–30%

2C	Severe: >30%

3	Subchondral collapse (crescent sign) without flattening

3A	Mild: < 15% of articular surface

3B	Moderate: 15–30%

3C	Severe: > 30%

4	Femoral head flattening

4A	Mild: <15% of surface and <2 mm depression

4B	Moderate: 15–30% of surface or 2–4 mm depression

4C	Severe: >30% of surface or >4 mm depression

5	Joint space narrowing with or without acetabular involvement

6	Advanced degenerative changes

**TASK：**

Determine presence/absence of femoral head lesion(s)，and identify whether it is unilateral or bilateral.

If positive:

a. Localize lesion(s) using topographic mapping.

b. Stage according to provide criteria with sub-classification.

c. Identify secondary degenerative changes.

If there are no characteristic findings, the absence of femoral head necrosis should be clearly stated.

**REPORTING SPECIFICATIONS:**

Binary lesion confirmation for each side with a confidence score.

If present, the specific Grade (including sub-classification) based on the requested criteria.

**Representative ID Radiology Description Examples**

The following de-identified radiology descriptions illustrate the text component used in the ID configuration. These descriptions were translated into English from the objective imaging-findings section of the original clinical reports. Patient identifiers, diagnostic conclusions, and staging labels were removed before model evaluation.

Example 1, pelvic radiograph:

Pelvic anteroposterior radiograph shows asymmetric bilateral femoral heads. The right femoral head is flattened and deformed, with shortening and thickening of the femoral neck. Patchy cystic low-density areas and irregular high-density areas are visible within the right femoral head. The right hip joint space is narrowed. The left femoral head morphology is preserved, with uneven bone density and small patchy low-density areas. Patchy sclerosis is visible at the left femoral head-neck junction. No obvious abnormality is seen in the bilateral sacroiliac joints or pubic symphysis.

Example 2, pelvic MRI:

Pelvic MRI shows asymmetric bilateral femoral heads. On T1-weighted imaging, linear low-signal intensity is visible along the margin of the left femoral head, with corresponding high-signal intensity on T2-weighted fat-suppressed imaging. Patchy low-signal intensity is seen in the left femoral head, with a small collapse-like depression in the superior femoral head contour. Edema-like signal is present around the left hip joint and adjacent soft tissue. The right femoral head and acetabulum show preserved alignment. The bilateral hip joint spaces are not obviously widened or narrowed.

## Supplementary Table S4. Performance metric comparison between MLLMs and DL models for ONFH.

| **Study** | **Model** | **Medical Imaging** | **AUC** | **Accuracy** | **Precision** | **Recall** | **F1 Score** |
| --- | --- | --- | --- | --- | --- | --- | --- |
| Michail *et al*.2024^26^ | VGG16 | MRI | 0.88 | 67% | 0.79 | 0.69 | 0.65 |
| Michail *et al*.2024^26^ | InceptionV3 | MRI | 0.75 | 73% | 0.75 | 0.72 | 0.72 |
| Michail *et al*.2024^26^ | Inception ResNetV2 | MRI | 0.77 | 73% | 0.77 | 0.71 | 0.71 |
| Xianyue *et al*.2023^14^ | Densenet | MRI | 0.89 | 88% | 0.85 | 0.85 | 0.85 |
| Our study | MLLMs-SI | MRI | 0.53 | 57% | 0.51 | 0.51 | 0.51 |
| Our study | MLLMs-ID | MRI | 0.91 | 77% | 0.71 | 0.69 | 0.70 |
| Our study | MLLMs-MI | MRI | 0.52 | 59% | 0.55 | 0.57 | 0.56 |

Legend: Performance metrics for MLLMs and published DL models.

Abbreviations: MLLMs, multimodal large language models; MRI, magnetic resonance imaging; AUC, area under the curve; SI, single image; ID, image plus radiology description; MI, multi-image; MIS1, multi-image setup 1; MIS2, multi-image setup 2.
